# Factors Influencing Psychological Distress in Caregivers of People with Dementia

**DOI:** 10.3390/healthcare13131582

**Published:** 2025-07-02

**Authors:** Nantiya Umpimai, Sopin Sangon, Patcharin Nintachan

**Affiliations:** 1 Master of Nursing Science Program in Psychiatric and Mental Health Nursing, Ramathibodi School of Nursing, Faculty of Medicine Ramathibodi Hospital, Mahidol University, Bangkok 10400, Thailand; nantiyaupm@gmail.com; 2 Ramathibodi School of Nursing, Faculty of Medicine Ramathibodi Hospital, Mahidol University, Bangkok 10400, Thailand; patcharin.nin@mahidol.ac.th

**Keywords:** caregivers, dementia, psychological distress, perceived caregiver burden, Thailand

## Abstract

**Background/Objectives:** This predictive research aimed to examine the influences of gender, perceived patients’ ability to perform activities of daily living, perceived impact of behavioral and psychological symptoms of dementia (BPSD), perceived caregiver burden, and perceived social support on the psychological distress of family caregivers of persons with dementia. **Methods**: The sample consists of 172 family caregivers of persons with dementia who received services at the outpatient department of a hospital in Bangkok and met the inclusion criteria. The research tools used in this study included a demographic questionnaire, the Kessler Psychological Distress Scale, the Barthel Activities of Daily Living Index, a Thai tool for assessing behavioral and psychological symptoms of dementia, the Thai Burden Interview for Caregivers of Patients with Chronic Illness, and Social Support Scale. The data were analyzed using descriptive statistics and multiple regression analysis. **Results**: The research findings revealed that 26.2% of the sample experienced psychological distress. Multiple regression analysis indicated that gender, perceived patients’ ability to perform activities of daily living, perceived impact of BPSD, perceived caregiver burden, and perceived social support could jointly explain 66.3% of the variance of psychological stress of family caregivers of persons with dementia (R^2^ = 0.663, F = 65.303, *p* < 0.001). The factors that significantly influenced psychological distress in family caregivers of dementia were perceived caregiver burden (β = 0.693, *p* < 0.001) and perceived impact of BPSD (β = 0.164, *p* < 0.01). **Conclusions**: The findings from this study can serve as a basis for developing strategies to reduce or prevent psychological distress in family caregivers of persons with dementia.

## 1. Introduction

Dementia is a general term encompassing several progressive diseases primarily affecting memory, cognitive abilities, and behavior [1]. Dementia is one of the leading causes of disability and dependency among older persons worldwide. It is estimated that around 55 million people globally have dementia [2]. Alzheimer’s disease is the most common form of dementia, contributing to 60–70% of cases [2]. In Thailand, the prevalence of dementia, as surveyed in 2014, was 8.1%. The highest prevalence is among the population aged 80 years and over [3]. Dementia affects people physically, mentally, socially, and economically [4,5,6]. It affects not only patients but also caregivers, family, and society as well [2]. As the disease advances significantly, the resulting impairments substantially disrupt an individual’s capacity to engage in daily activities and require care from caregivers [1,7]. Because of the dependency of individuals with dementia, caregivers caring for people with dementia are very important people.

The family caregiver is a family member, such as a father, mother, husband, wife, child, or grandchild, who provides care and assistance to a family member who is unable to care for himself or herself due to illness or disability without receiving any compensation for the care [8,9]. In Thailand, family caregivers, particularly daughters and daughters-in-law, play a crucial role in caring for individuals with dementia [10]. Cultural values such as “katanyu kataweti” emphasize the importance of honoring and repaying parents for their support. The shortage of long-term care facilities further increases the reliance on family caregivers. These caregivers frequently perform their roles without financial compensation and encounter substantial challenges [11,12]. Because of the symptoms and dependency of individuals with dementia, providing care for them is challenging, time consuming, and requires long periods of care, causing caregivers to be physically, mentally, socially, and economically affected [13,14,15,16]. It was found that dementia caregivers tended to experience more psychological distress, such as anxiety [17,18] and depression [17,18,19,20], than non-dementia caregivers [20,21] and the general population [22]. Nevertheless, they also experience positive aspects associated with caregiving.

Psychological distress is a state of mental discomfort that is often expressed through feelings of nervousness, hopelessness, restlessness/fidgeting, a sense of putting in great effort, depression, and worthlessness [23]. A study by Shikimoto et al. [15] found that 17.7% of the caregivers of people with dementia had psychological distress. Psychological distress of caregivers of people with dementia may put them at risk of illnesses, affecting their quality of life and their care efficiency [24,25,26]. A review of previous studies revealed that factors significantly predicting psychological distress among caregivers of people with dementia were behavioral and psychological symptoms of dementia [16,17,27], coping strategies [28], perceived caregiver burden [25], and quality of life [29], the mental state of people with dementia [17,27], the ability to perform basic activities of daily living [17,30], the relationship of caregivers and patients as parents and children [15], the duration of care [15,17], age of caregivers [30], and social support [30].

In Thailand, research examining the mental health effects of caregiving for people with dementia has mostly focused on depression [10,19,31,32,33]. Given the diverse impacts of caring for individuals with dementia, examining psychological distress and mental discomfort with various symptoms offers a more comprehensive understanding of the psychological effects on caregivers. The predictive research on the psychological effects of caregiving for individuals with dementia in Thailand is also limited [17,27,34,35,36]. Studying the factors that influence psychological distress in caregivers of individuals with dementia is critical to inform the development of strategies for reducing distress and enhancing the mental health of caregivers, who play a vital role in supporting those with dementia.

**Figure 1:**
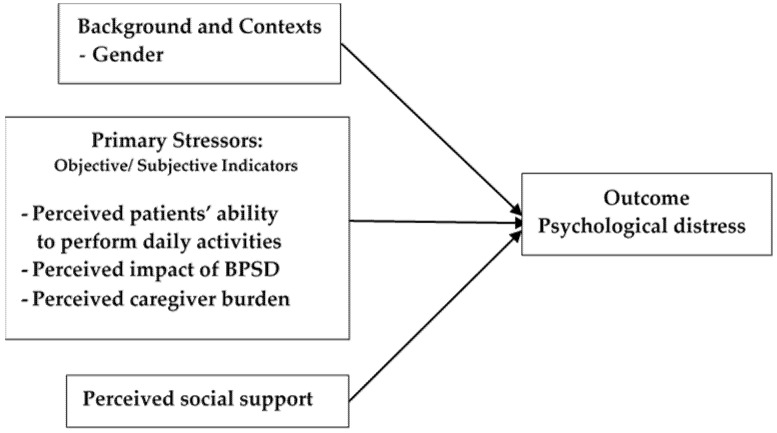
Conceptual framework of the study.

The conceptual framework used in this study was guided by the Stress Process Model of Pearlin et al. [8] and the literature review. This model identified sources of stress and outcomes specific to caregiving [25]. Consistent with the Stress Process Model of Pearlin et al. [8], this study investigated factors (gender, the perceived patients’ ability to perform daily activities, the perceived impact of BPSD, perceived caregiver burden, and perceived social support) that may be associated with psychological distress (outcome of stress). Although the Stress Process Model of Pearlin et al. [8] mentioned social support as a mediator, social support can independently affect stress outcome, such as psychological distress [37]. Therefore, social support was studied as a factor influencing psychological distress (Figure 1).

Gender

According to the Stress Process Model of Pearlin et al. [8], the characteristics or background of caregivers (e.g., gender) influence stress outcome, such as psychological distress. Previous studies revealed that female caregivers tended to have more psychological distress than male caregivers [16,38]. This is possibly because female caregivers spend more time providing care than male caregivers. Female caregivers are more likely to provide assistance with personal care, such as bathing, than male caregivers, and female caregivers often have to play multiple roles, such as a wife and mother, which can lead to role stress [39]. However, some studies found that the psychological distress of male and female caregivers of people with dementia was not different [13]. A study by Sornchai et al. [40] also showed that gender was not associated with depression among female caregivers of people with dementia. However, some studies found that the gender of caregivers was related to psychological distress among female caregivers of people with dementia [16,38].

Perceived patients’ ability to perform daily activities

The ability to perform daily activities of people with dementia may be impaired when there has been substantial progression of disease [1,7]. It was found that dementia caregivers tended to provide more assistance to the patients in performing daily activities than non-dementia caregivers [21]. According to the Stress Process Model of Pearlin et al. [8], the more dependent the patients are, the more intensive and difficult it will be for caregivers. Attempting to respond to the demands of the patient’s daily living activities often results in psychological distress for the caregivers. This is consistent with a previous study which found that the caregivers of people with dementia with low abilities to perform activities of daily living were likely to have high psychological distress [38,41], and the ability to perform basic activities of daily living could predict psychological distress among caregivers of people with dementia [30].

Perceived impact of BPSD

Behavioral and psychological symptoms of dementia (BPSD) refer to neuropsychiatric symptoms in individuals with dementia [42]. BPSD involves symptoms such as psychotic symptoms, mood symptoms, and sleep symptoms [16,27,42]. More than 90% of people with dementia will exhibit at least one BPSD at some point in the course of their illness [4]. BPSD affects not only the patients themselves but also their caregivers [16]. Research has shown that these symptoms can significantly contribute to the psychological distress experienced by caregivers of people with dementia [16,17,38,41].

Perceived caregiver burden

Perceived caregiver burden refers to the perception of caregivers regarding their emotional, physical, and financial well-being as being negatively affected by caring for a family member with dementia [43]. Perceived caregiver burden is a source of stress [8]. Caregivers of individuals with dementia who encounter challenging caregiving situations and perceive these situations as burdensome are likely to develop psychological distress. This aligns with previous research, which has shown that perceived caregiver burden can predict psychological distress among those caring for people with dementia [25].

Perceived social support

Social support is defined as a composite concept including (1) attachment/intimacy, (2) social integration, (3) nurturance, (4) reassurance of worth, and (5) availability of assistance. Perceived social support may come from an individual’s interactions with their social environment. Previous studies have shown that social support is negatively associated with psychological distress among caregivers of people with dementia [16,44,45] and can even predict levels of psychological distress in this group [30]. When caregivers encounter challenging or stressful caregiving situations and believe they have the resources to manage them, they experience reduced stress and lower levels of psychological distress. Previous studies indicate that caregivers of individuals with dementia who perceive high levels of social support tend to experience less psychological distress [46].

The purpose of this study was to examine the ability of gender, perceived patients’ ability to perform daily activities, perceived impact of behavioral and psychological symptoms of dementia (BPSD) on family caregivers, perceived caregiver burden, and perceived social support in jointly predicting psychological distress among family caregivers of people with dementia.

## 2. Materials and Methods

### 2.1. Study Design

This study used a predictive design.

### 2.2. Study Participants

Participants were family caregivers of people diagnosed with dementia receiving services at the outpatient department of a hospital in Bangkok, based on the following inclusion criteria: (1) being a primary caregiver who has provided care for the person with dementia for more than 6 months, has a relationship as a father, mother, husband, wife, child, grandchild, son-in-law, daughter-in-law, or relative, and has lived with the patient and has helped with activities of daily living without being paid, (2) aged 18 years or older, both male and female, (3) not having cognitive impairments in those who were older than 60 years old. They all were tested by the Set Test [47], with scores higher than or equal to 25 included in the study, and (4) being able to understand and communicate well in Thai.

The sample size was calculated using Cohen’s power analysis concept [48]. The power of the test was set at 0.80, and the statistical significance was 0.05. There were 5 predictive variables in this study. The effect size was determined based on the literature review [13,15,16,17,25,30,41,46]. This study’s effect size was small, with a value set at 0.08. The data were analyzed using multiple regression analysis. The sample size was calculated using the G*Power 3.1.9.7 program [49], and a sample size of 166 people was obtained. To prevent incomplete data, the sample size was increased by another 10%, resulting in a total sample of 183 people. From the data collection, 172 participants volunteered to participate in the study, which was sufficient for the sample size calculation of this study.

### 2.3. Research Instruments

#### 2.3.1. The Set Test

The Set Test was developed by Isaacs and Kennie [47]. It was used to screen the memory and cognition of the caregivers of people with dementia over 60 years. The test consists of 4 categories: colors, animals, fruits, and cities. Each category has 10 names. A correct answer will obtain 1 point. Each category has a full score of 10 points. The total score for all categories is between 0 and 40 points. Those scoring 25 points or more were considered to have passed the sample selection criteria.

#### 2.3.2. The Demographic Questionnaire

The researcher developed the questionnaire, which consists of questions about personal information. The questions regarding gender, marital status, education level, occupation, adequacy of income, and the relationship between the caregiver and the patient were closed-ended questions with options. As for age, income, and time spent caring for the patient, these were short-answer questions for the participants to provide their information.

#### 2.3.3. Kessler Psychological Distress Scale 6 Items (K6), Thai Version

The Kessler Psychological Distress Scale 6 Items (K6) Thai version was used to measure the psychological distress of family caregivers. The K6 was developed by Kessler et al. [50], translated into the Thai language using the back translation technique, and validated with pilot subjects by Suraaroonsamrit and Arunpongpaisal [51]. The K6 was designed to assess the frequency of non-specific psychological distress within a particular reference period [50]. It consists of 6 items. Participants were asked to report how often they felt the following emotions over the past 30 days: (1) nervous, (2) hopeless, (3) restless or fidgety, (4) that everything was an effort, (5) depressed, and (6) worthless. Their responses were recorded on a 5-point Likert scale, where “none of the time” was scored as 0 and “all of the time” was scored as 4. The items are summed to calculate the total psychological distress score. The total score is between 0 and 24 points. A high total score indicates a high level of psychological distress. According to the study by Suraaroonsamrit and Arunpongpaisal [51], a cut-off point in the Thai population is 4. Therefore, scores of 0–3 points indicate no psychological distress [51], and scores of 4–24 points indicate psychological distress [51]. In this study, from a total sample of 172 participants, the Cronbach’s alpha reliability coefficient was found to be 0.79.

#### 2.3.4. Barthel Activities of Daily Living Index, Thai Version

This tool was developed by Barthel and Mahoney [52] to assess the ability to perform activities of daily living of people with dementia and modified by Jitapunkul et al. [53]. The responses are on a 2-point rating scale (2 items), a 3-point rating scale (6 items), and a 4-point rating scale (2 items). The total score is between 0 and 20 points. A high total score means that the person can perform basic activities of daily living very well. The interpretation of scores is as follows: scores of 0–8 indicate a low level of the ability to perform basic activities of daily living, scores of 9–11 indicate a moderate level of the ability to perform basic activities of daily living, and scores of 12–20 indicate a normal level of the ability to perform basic activities of daily living [54]. In this study, from a total sample of 172 participants, the Cronbach’s alpha reliability coefficient was found to be 0.95.

#### 2.3.5. Behavioral and Psychological Symptoms of Dementia–Thai (BPSD-T)

This tool was developed by Phannarus et al. [42] to assess behavioral and psychological symptoms of dementia (BPSD) in people with dementia and the impact of BPSD on their caregivers. The BPSD-T comprises 14 items asking whether the patient had any of the following BPSD symptoms in the past month, for example, “aggressive behavior”, “irritability”, and “delusion” [42]. It was scaled as “presence (0)” or “absence (1)” of symptoms. If the participant reported that the person with dementia had a specific symptom, then the additional question regarding the impact of the symptoms on the caregiver is evaluated. The responses are on a 4-point rating scale (1 = no impact on caregiver; 2 = little impact; 3 = some impact but still bearable; 4 = a lot of impact which cannot be handled). The possible total score is in the range of 0–56 points. For participants who do not experience all 14 BPSD symptoms, the score will be 0. A high total score indicates a high level of perceived impact of BPSD on the caregiver. In this study, from a total sample of 172 participants, the Cronbach’s alpha reliability coefficient was found to be 0.78.

#### 2.3.6. Thai Burden Interview for Caregivers of Patients with Chronic Illness

This tool was developed by Zarit and Zarit [43] to assess caregivers’ burden on patients with chronic illness. The Burden Interview was translated into the Thai language using a blind back translation method and then tested for validity and reliability in Thai subjects by Toonsiri et al. [55]. The Thai Burden Interview had good validity and reliability. There are 22 items. Caregivers were asked to indicate how often they experience the feeling in each item when taking care of their relatives, for example: “Do you feel that your relative asks for more help than (s)he needs?”, “Do you feel that because of the time you spend with your relative that you don’t have enough time for yourself?” The responses are on a 5-point rating scale from 0 (never) to 4 (nearly always). Total scores are in the range of 0–88 points. A high total score indicates a high burden. In this study, from a total sample of 172 participants, the Cronbach’s alpha reliability coefficient was found to be 0.92.

#### 2.3.7. Social Support Scale, Thai Version

The Social Support Scale was developed by Weinert [56] to assess respondents’ perceived social support. It was translated into the Thai language by Thungmiphon [57]. It consists of 15 items, for example, “there is someone I feel close to who makes me feel secure.”, and “there are people who are available if I need help over an extended period of time”. The item responses range from 1 (strongly disagree) to 7 (strongly agree). Total scores range from 15 to 105 points, with higher total scores indicating higher perceived social support [56]. The interpretation of scores can be divided into the following levels: scores of 15–45 indicate low perceived social support, 46–75 indicate moderate perceived social support, and 76–105 indicate high perceived social support [57]. The Social Support Scale (Thai version) has been used to measure perceived social support in Thai family caregivers. It has been tested for content validity by experts and has good reliability [58,59]. In this study, from a total sample of 172 participants, the Cronbach’s alpha reliability coefficient was found to be 0.90.

### 2.4. Human Subjects Protection

This research complied with human research ethics and was approved by the Human Research Ethics Committee, Faculty of Medicine, Ramathibodi Hospital, Mahidol University (Reference No. MURA 2022/186). It also received permission to collect data from the hospital director and was approved by the hospital’s Human Research Ethics Committee, where the data collection was performed. Regarding data collection, the researcher clarified the objectives and data collection processes to the participants and emphasized that they had the right to decide whether to consent or refuse to participate and withdraw from the research at any time, without any consequences. Once the participants consented and voluntarily participated in the study, they were asked to sign the consent form. The data obtained from the research were confidential and used only for research purposes. The data were stored in secure locations, and access was strictly controlled to prevent unauthorized use. The research findings were presented without disclosing the participants’ real names or surnames.

### 2.5. Data Collection

The data collection was administered after receiving permission from all committees and the hospital mentioned previously. The data were collected between July 2022 and February 2023. The research project was promoted and publicized at the dementia clinic of the outpatient department, and the researcher collaborated with the head nurse and staff nurse in the dementia clinic to publicize the project regarding the study’s objectives, inclusion criteria, data collection procedures, potential risks, and benefits. The potential participants were approached by researchers. The potential participants who were older than 60 years old were tested by the Set Test. If they had scores higher than or equal to 25, they were included in the study. Participants were given time to decide whether to participate in the study. They were asked to sign a consent form if they decided to participate.

### 2.6. Data Analysis

The data were analyzed using SPSS version 29.0.1.0 (IBM Corp., Armonk, NY, USA). Descriptive statistics were employed to analyze the demographic information of family caregivers of people with dementia and the variables in this study. Multiple regression analysis was used to analyze the ability of gender, perceived patients’ ability to perform activities of daily living, perceived impact of BPSD, perceived caregiver burden, and perceived social support to predict the psychological distress of family caregivers of people with dementia. The significance level was set at 0.05. For gender, a nominal scale was adjusted to be a dummy variable: 1 for female and 2 for male, to enable multiple linear regression analysis. Before the data analysis, assumptions for multiple regression analysis, including normality, linearity, multicollinearity, and homoscedasticity, were tested. Normality was assessed using skewness and kurtosis values. The acceptable ranges for a normal distribution are skewness values between −3 and +3 and kurtosis values between −10 and +10 [60]. In this study, the skewness values ranged from −1.31 to 1.98, while the kurtosis values ranged from 0.45 to 5.24, indicating that the normality assumption was met. Furthermore, residual analysis was performed [61], and the histogram of the standardized residual revealed a normal curve, confirming that the normality assumption was met. Linearity was assessed using scatterplots, which showed a straight line, confirming linear relationships between the predictors and the dependent variables [62]. Multicollinearity was examined using variance inflation factors (VIF) and tolerance levels. A VIF exceeding four or a tolerance below 0.25 may indicate the presence of multicollinearity [63]. In this study, the VIF ranged from 1.01 to 1.38, and the tolerance levels ranged from 0.72 to 0.99, suggesting that there was no multicollinearity. Homoscedasticity was tested using a scatter plot of standardized residuals against standardized predicted values [63].

## 3. Results

### 3.1. The Demographic Information of the Samples

Of 172 family caregivers of people with dementia, the majority were female (73.8%). The mean age was 53.7 (SD = 11.6). They were mostly married (58.7%) and earned a bachelor’s degree or higher (56.4%). About thirty percent of the participants were unemployed or retired (30.8%). The average monthly income was 26,890.7 baht/month (Min = 0, Max = 250,000, Mode = 20,000 baht/month). Most of them had adequate income (66.9%). They were mainly related to the patients as children (61.6%) and spent an average of 15.5 h/day (SD = 7.1) caring for them.

### 3.2. Descriptive Statistics of Major Variables

The perceived patients’ ability to perform daily activities had a mean score of 15.22 (SD = 6.27). The family caregivers reported that most people with dementia under their care had a normal ability to perform daily activities (78%). The mean score of perceived impact of BPSD was 5.50 (SD = 5.90). The average total score of perceived caregiver burden was 14.26 (SD = 12.72). In total, 91.3% of family caregivers reported having high perceived social support. The mean score of perceived social support was 89.69 (SD = 12.18). The mean score of psychological distress was 2.40 (SD = 2.95). Please see Table 1 for details. Approximately 26% of family caregivers in this study reported having psychological distress. Please see Table 2 for details.

### 3.3. Correlations Between Study Variables

Table 3 presents relationships between study variables. Results show that the perceived patients’ ability to perform daily activities and perceived social support significantly and negatively correlated with psychological distress (rho = −0.31, *p* < 0.01, rho = −0.27, *p* < 0.01, respectively). The perceived impact of BPSD and perceived caregiver burden significantly and positively correlated with psychological distress (rho = 0.48, *p* < 0.01, rho = 0.78, *p* < 0.01, respectively). Gender was not correlated with psychological distress.

### 3.4. Factors Predicting Psychological Distress in Family Caregivers of People with Dementia

The analysis results of the ability to predict psychological distress of family caregivers of people with dementia revealed that gender, perceived patients’ ability to perform daily activities, perceived impact of BPSD, perceived caregiver burden, and perceived social support jointly explained 66.3% of the variance in family caregivers’ psychological distress (R^2^ = 0.663, F = 65.303, *p* < 0.001). The factors that significantly influenced the psychological distress of family caregivers of people with dementia included perceived impact of BPSD (β = 0.164, *p* < 0.01) and perceived caregiver burden (β = 0.693, *p* < 0.001) (Table 4).

## 4. Discussion

The findings indicated that gender, perceived patients’ ability to perform daily activities, perceived impact of BPSD, perceived caregiver burden, and perceived social support jointly explained 66.3% of the variance in family caregivers’ psychological distress. Factors that significantly influence psychological distress are the perceived impact of BPSD and the perceived caregiver burden. Perceived caregiver burden is the most significant influencing factor on the psychological distress of family caregivers of people with dementia, followed by perceived impact of BPSD. Gender, perceived patients’ ability to perform daily activities, and perceived social support did not significantly influence psychological distress in family caregivers of people with dementia.

### 4.1. Perceived Caregiver Burden

The caregiver’s burden is how caregivers feel their emotional or physical health, social life, and finances are affected by caring for their dementia relative [43]. The study result indicates that perceived caregiver burden had the most significant influence on the psychological distress of caregivers of people with dementia (β = 0.693, *p* < 0.001). This indicates that the family caregivers of people with dementia who perceived a high burden had a high chance of having psychological distress. This result is consistent with previous studies, which revealed that perceived caregiver burden could predict the psychological distress of family caregivers of people with dementia [13,25]. This is consistent with the Stress Process Model of Pearlin et al. [8], stating that sources of stress can lead to the outcome of stress among family caregivers. In this study, burden as the source of stress can significantly influence psychological distress, which is the outcome of stress, of family caregivers of people with dementia. It can be explained that burden is the perception of negative impacts on the body, mind, emotion, society, and economy of family caregivers resulting from caring for patients. In addition, dependent patients require continuous and long-term care. This type of care may cause family caregivers to feel burdened and experience psychological distress. Therefore, healthcare providers should pay more attention to reducing the burden perceived by family caregivers of people with dementia.

### 4.2. Perceived Impact of BPSD

In this study, the perceived impact of BPSD significantly predicts the psychological distress of family caregivers of people with dementia (β = 0.164, *p* < 0.01). This indicates that the family caregivers of people with dementia who highly perceived the impact of BPSD were at high risk of developing psychological distress. It is consistent with a previous study, which indicated that the perceived impact of BPSD influenced the psychological distress of family caregivers of people with dementia [16,17,38,41]. It can be explained that BPSD can occur among people with dementia. BPSD includes symptoms such as aggression, irritability, delusion, apathy, repetitive behavior, depression, anxiety, and hallucination. Each demented person may have different symptoms. BPSD may affect family caregivers if they are unable to manage the BPSD, leading to a perceived impact of BPSD. Moreover, BPSD are classified as sources of stress based on the patient’s health and behavior [8]. When family caregivers are exposed to something stressful, it can negatively impact them, causing them to become stressed or experience psychological distress as a result of caring for people with dementia who are exhibiting BPSD. This aligns with the Stress Process Model of Pearlin et al. [8], stating that sources of stress can lead to the outcome of stress among family caregivers. Congruent with Pearlin et al.’s Stress Process Model, this study found that the perceived impact of BPSD on people with dementia influenced the psychological distress of family caregivers of people with dementia. Therefore, interventions to reduce the perceived impact of BPSD on family caregivers should be focused on.

### 4.3. Gender

Gender did not significantly influence psychological distress in family caregivers of people with dementia. The finding is inconsistent with previous studies, which found that female family caregivers of people with dementia had more psychological distress than male family caregivers [38], and female gender was associated with psychological distress in dementia family caregivers [16]. However, it is consistent with some previous studies, which found that the psychological distress of male and female family caregivers of people with dementia was not different [13], and gender was not associated with depression in family caregivers of people with dementia [40]. According to Thai culture, caring for elderly parents with dementia is a strong component of familial duty. The concept of “katanyu” in Thai society emphasizes gratitude and respect toward parents, motivating children to care for them in their old age [12]. Caregiving is a repayment for the upbringing received, reinforcing familial bonds [12]. Therefore, both female and male children have responsibilities for caring for their parents with dementia. In this study, most of the samples also included the patients’ children (61.60%). This might be the reason why gender did not influence the psychological distress of family caregivers of people with dementia in this study.

### 4.4. Perceived Patients’ Ability to Perform Activities of Daily Living

The patients’ ability to perform basic activities of daily living, as perceived by family caregivers, was significantly and negatively correlated with psychological distress (rho = −0.31, *p* < 0.01). However, it had an insignificant influence on the psychological distress of family caregivers of people with dementia (β = −0.062, *p* = 0.192). This result is inconsistent with some studies that found that the ability to perform basic activities of daily living of people with dementia could predict the psychological distress of family caregivers of people with dementia [30]. However, the result is consistent with a previous study that showed that the ability to perform basic activities of daily living, as perceived by family caregivers of people with dementia, could not predict the psychological distress of family caregivers of people with dementia [17]. The symptoms of dementia with progressive cognitive decline can cause a decrease in the patients’ ability to perform daily activities. Due to the patients’ and their care needs, the amount and difficulty of caregiving can increase accordingly. When family caregivers try to meet the needs of each activity related to the patient’s daily life, it often causes stress for the family caregivers. The Stress Process Model of Pearlin et al. [8] explained that the symptoms of people with dementia with progressive cognitive decline can cause a decrease in the patients’ ability to perform daily activities. The failure of the patients’ ability to perform daily activities, as perceived by family caregivers, to display a statistically significant influence on psychological distress is noteworthy. This may result from the recruitment of the participants from only one tertiary hospital in Bangkok, leading to the invariance of this variable’s data. Most people with dementia in this study were perceived by their family caregivers to have the normal ability to perform daily living activities (78%), obscuring the influence of the patients’ ability to perform daily activities on psychological distress in this study. Further research should consider collecting data from family caregivers of people with dementia with a wide range of abilities to perform daily activities.

### 4.5. Perceived Social Support

Perceived social support was significantly and negatively correlated with psychological distress (rho = −0.27, *p* < 0.01). However, the influence of perceived social support was not strong enough to exert a predictive role on the psychological distress of family caregivers of people with dementia (β = 0.062, *p* = 0.182). The result is inconsistent with previous studies, which found that perceived social support could predict the psychological distress of family caregivers of people with dementia [30,64,65]. However, the result is consistent with a previous study, which revealed that perceived social support did not predict the psychological distress of family caregivers of people with dementia [66]. The result of this study indicates that family caregivers who perceived higher social support did not demonstrate lower psychological distress. This finding is noteworthy as it underscores the limited predictive power of perceived social support regarding psychological distress in this study. The explanation may be related to the complex and multifaceted relationships between social support and psychological distress among family caregivers of individuals with dementia. This may be attributed to various factors, including the type of support received [66], the quality of support [65], individual differences [12,67], and the role of social support [68]. However, the Stress Process Model of Pearlin et al. [8] explained social support function as a mediator between stressor and psychological distress, but was not investigated in this study. Further research is recommended to explore the mediating effect of social support.

## 5. Conclusions

This study reveals that the perceived impact of BPSD and perceived caregiver burden significantly predict the psychological distress of people with dementia. The significance of the perceived impact of BPSD on family caregivers’ psychological distress necessitates the implementation of targeted interventions. These interventions should focus on reducing the perceived impact of BPSD. Additionally, perceived caregiver burden experienced by family caregivers of people with dementia significantly impacts their psychological distress. Research suggests that implementing targeted interventions to mitigate perceived caregiver burden can help reduce psychological distress.

This study has some limitations. First, it utilized a cross-sectional design. While this approach was suitable for the study, it restricted the ability to interpret the findings beyond the associations between the measured variables, such as burden and psychological distress. Therefore, a longitudinal design would benefit future research, allowing for a better examination of the causal relationships between these variables. Second, this study gathered data from urban areas, which limits the generalizability of the findings to family caregivers in rural areas of Thailand. Third, the restriction of cognitive functioning assessments to individuals aged 60 and older may limit the generalizability of the findings to family caregivers who possess different characteristics from those represented in the study population.

## Figures and Tables

**Table 1:** Descriptive statistics of study variables (*n* = 172).

Variables	Possible Range	Actual Range	Mean	SD
Perceived patients’ ability to perform daily activities	0–20	0–20	15.22	6.27
Perceived impact of BPSD	0–56	0–32	5.55	5.90
Perceived caregiver burden	0–88	0–61	14.26	12.72
Perceived social support	15–105	43–105	89.69	12.18
Psychological distress	0–24	0–14	2.40	2.95

**Table 2:** Numbers and percentages of family caregivers of people with dementia by psychological distress (*n* = 172).

Psychological Distress	*n*	Percentage
No psychological distress (K6 = 0–3 score)	127	73.8
Having psychological distress (K6 = 4–24 score)	45	26.2

**Table 3:** Correlation metrics of the study ariables (*n* = 172).

Variables	Gender ^T^	Perceived Patients’ Ability to Perform Daily Activities	Perceived Impact of BPSD	Perceived Caregiver Burden	Perceived Social Support	Psychological Distress
Gender	1					
Perceived patients’ ability to perform Daily activities	0.07	1				
Perceived impact of BPSD	0.05	0.00	1			
Perceived caregiver burden	0.03	−0.25 *	0.45 **	1		
Perceived social support	−0.06	0.09	−0.13 *	−0.19 *	1	
Psychological distress	0.08	−0.31 **	0.48 **	0.78 **	−0.27 **	1

All correlation coefficients are Spearman Rho except ^T^ is point-biserial; * correlation is significant at the 0.05 level (two-tailed); ** correlation is significant at the 0.01 level (two-tailed).

**Table 4:** Multiple linear regression of predictors of psychological distress in family caregivers of people with dementia (*n* = 172).

Variables	b	SE	β	*t*	*p*-Value
Gender	0.310	0.303	0.046	1.022	0.308
Patients’ ability to perform daily activities	−0.029	0.022	−0.062	−1.310	0.192
Perceived impact of BPSD	0.082	0.026	0.164 **	3.210	0.002
Perceived caregiver burden	0.161	0.012	0.693 ***	13.048	0.000
Perceived social support	−0.015	0.011	−0.062	−1.341	0.182

Constant = 1.359; R = 0.814; R^2^ = 0.663; Adjusted R^2^ = 0.653; R^2^_change_ = 0.004; Overall F_(5166)_ = 65.303; *p* < 0.001; ** *p* < 0.01; *** *p* < 0.001.

## Data Availability

The data presented in this study are unavailable due to privacy and ethical restrictions.
